# Diffusion Tensor Imaging Magnetic Resonance Imaging Assessment in a Clinical Trial of Autologous Dendritic Cell Transfer for Diabetic Kidney Disease: A Molecular Approach

**DOI:** 10.3390/diseases13050159

**Published:** 2025-05-19

**Authors:** Ernaldi Kapusin, Aditya Pratama Lokeswara, Yudo Rantung, Bhimo Aji Hernowo, Jonny Jonny, Chrismis Novalinda Ginting, Terawan Agus Putranto

**Affiliations:** 1 Faculty of Medicine, Dentistry, and Health Sciences, Universitas Prima Indonesia, Medan 20118, Indonesia; ernaldirspad@gmail.com (E.K.); hernowobhimo@gmail.com (B.A.H.); jonny@unprimdn.ac.id (J.J.); chrismis@unprimdn.ac.id (C.N.G.); 2 Department of Radiology, Gatot Soebroto Army Hospital, Jakarta 10410, Indonesia; lokeswaraaditya@gmail.com (A.P.L.); yudorantung@gmail.com (Y.R.); 3 Indonesia Army Cellcure Center, RSPAD Gatot Soebroto, Jakarta 10410, Indonesia; 4 Faculty of Military Medicine, Indonesia Defense University, Bogor 16810, Indonesia; 5 Nephrology Division, Department of Internal Medicine, Gatot Soebroto Army Hospital, Jakarta 10410, Indonesia; 6 Faculty of Medicine, National Development University “Veteran” Jakarta, Jakarta 12450, Indonesia

**Keywords:** diabetic kidney disease, autologous dendritic cells, fractional anisotropy, Matrix Metalloproteinase-9, Intercellular Adhesion Molecule-1

## Abstract

Background: Continuous rise of type 2 diabetes mellitus (T2DM) global prevalence, has led to a subsequent increase in the prevalence of diabetic kidney disease (DKD). DKD is associated with higher levels of inflammation and impaired kidney function. Many patients do not receive adequate treatment for this condition. This research aims to evaluate the therapeutic impact of autologous dendritic cell transfer by examining its effects on renal microstructural changes as assessed through Diffusion Tensor Imaging (DTI) MRI, alongside the analysis of key inflammatory biomarkers, namely Matrix Metalloproteinase-9 (MMP-9) and Intercellular Adhesion Molecule-1 (ICAM-1). Methods: A clinical trial with an open-label design was performed with 25 DKD patients receiving outpatient care at Gatot Soebroto Army Hospital. Each participant was administered a single injection of autologous dendritic cells. Evaluations were conducted both prior to and one month following the treatment. The primary measurements included Diffusion Tensor Imaging (DTI) MRI-derived Fractional Anisotropy (FA) scans and the inflammatory biomarker MMP-9. Results: A notable increase in FA was observed, rising from 242.57 ± 63.97 at baseline to 305.61 ± 152.32 one month after the dendritic cell injection. However, there were no significant changes in MMP-9 and ICAM-1 levels. Additionally, a negative correlation was found between FA and MMP-9 (r = −0.324, *p* = 0.025). Conclusion: The transfer of autologous dendritic cells significantly enhanced FA, which correlates with a reduction in the inflammatory biomarker MMP-9, suggesting a potential impact on renal repair in DKD.

## 1. Introduction

Diabetes mellitus (DM) global prevalence is estimated at 9.3%, with projections indicating an increase to 10.2% by 2030 and 10.9% by 2045 [1]. Among individuals with type DM, diabetic kidney disease (DKD) is present in about 27% of cases, and those affected by DKD have a mortality rate 18.3 times greater than those without diabetes [2].

Inflammation is a crucial factor in the pathophysiology DKD, where immune cell activation leads to the production of pro-inflammatory cytokines. Key biomarkers, including matrix metalloproteinase 9 (MMP-9) and intercellular adhesion molecule (ICAM-1), are involved in driving kidney damage through inflammatory processes and fibrosis [3,4,5]. While these biomarkers are well established in the disease process, their direct correlation with non-invasive imaging biomarkers, particularly in the context of immunotherapy, remains underexplored

Diffusion tensor imaging (DTI) has become a valuable non-invasive method for evaluating kidney microstructure, offering insights into tissue integrity by measuring mean diffusivity (MD) and fractional anisotropy (FA) [6]. DTI has gained recognition as a valuable imaging technique for assessing kidney inflammation and fibrosis by identifying structural changes at the microstructural level. DTI utilizes the diffusion properties of water molecules to map tissue microstructure, with FA serving as a key metric. FA has shown sensitivity to a variety of kidney diseases, including chronic kidney disease (CKD) and glomerulonephritis, by detecting subtle alterations in kidney tissue integrity [6,7]. Recent studies have highlighted the growing importance of DTI in evaluating early-stage kidney damage and monitoring the progression of renal diseases, particularly in the context of DKD [8]. FA values correlate closely with renal function and pathological changes, offering a promising method for monitoring CKD progression [8].

Autologous dendritic cells, derived from the patient’s own immune system, have gained attention as a promising therapeutic option in modulating the immune response in various inflammatory conditions [9,10,11,12]. These cells can be genetically modified outside the body to enhance their functions on immune-regulatory before being reintroduced into the patient’s system. This process helps promote immune tolerance and decrease inflammation [13]. The use of autologous dendritic cells in therapy has shown considerable promise in reducing inflammation and fibrosis in various medical conditions, making them a highly potential treatment strategy for managing diabetic kidney disease (DKD).

Despite the potential of using autologous dendritic cells to modulate inflammation in diabetic kidney disease (DKD), and the integration of DTI MRI for monitoring, this approach has not been thoroughly explored. This study seeks to address this gap by assessing the potency of autologous dendritic cell transfer on renal inflammation and fibrosis, utilizing DTI MRI and inflammatory biomarkers (MMP-9 and ICAM-1) as key indicators of therapeutic efficacy.

## 2. Materials and Methods

### 2.1. Study Design

This investigation utilized a quasi-experimental design, specifically employing a one-group pre-test post-test methodology. The study was carried out at the Army Central Hospital, and ethical approval was obtained from the Ethics Committee of Gatot Soebroto Army Central Hospital (Ethical Clearance No. 110/VIII/KEPK/2024, dated 23 August 2024). All participants provided written informed consent prior to their involvement. The clinical trial is registered with ClinicalTrials.gov under the registration number NCT06866158, initially submitted on 22 February 2025. During the preparation of this manuscript, the authors utilized ChatGPT 4o for language enhancement and proofreading purposes. However, the authors have thoroughly examined and refined the output, taking full responsibility for the accuracy and integrity of the content presented in this publication.

### 2.2. Research Subject

**Figure 1:**
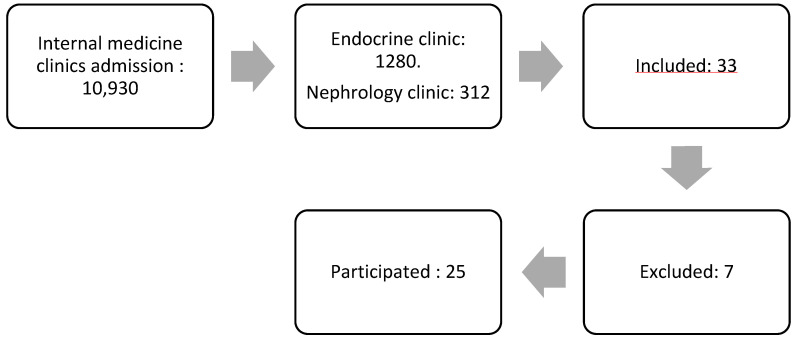
The flow of subject selection.

The study participants were patients diagnosed with diabetic kidney disease (DKD) from the internal medicine polyclinic at the Army Hospital. A nonprobability sampling method was utilized for participant selection. Figure 1 demonstrates the subject selection process. The initial pool comprised 10,930 patients from the internal medicine polyclinic. After narrowing down the selection, 1280 patients were from the Endocrine Clinic, and 312 were from the Kidney Clinic. A total of 33 patients expressed interest in participating, but seven were excluded, leaving 25 patients who met the inclusion criteria and proceeded with the study.

Patients were excluded from the study if they had undergone immunosuppressive treatment within the last four weeks, had other kidney diseases, were diagnosed with conditions causing proteinuria, had other types of DM, were pregnant, required oxygen supplementation, were undergoing cancer hormone therapy, history of thromboembolism, obesity with a BMI exceeding 40 kg/m^2^, or uncontrolled hypertension (systolic > 180 mmHg or diastolic > 100 mmHg).

The sample size for this study was determined utilizing the G*Power version 3.1.9.7 software, applying a two-tailed *t*-test approach. The calculation considered an effect size of 0.8, a significance level (α) of 0.05, and a statistical power of 0.95. Based on these parameters, the minimum required sample size was determined to be 23 participants to ensure sufficient statistical strength.

### 2.3. Research Procedure

The study procedure included several key steps. Initially, subjects were prepared for baseline measurements, which involved blood sampling to assess the biomarkers MMP-9 and ICAM-1, as well as the generation of autologous dendritic cells. Following this, subjects underwent Diffusion Tensor Imaging (DTI) MRI examinations of the kidneys.

The MMP-9, ICAM-1, and DTI MRI examinations were performed at two time points: before the transfer of autologous dendritic cells and four weeks following the treatment.

### 2.4. Study Product Generation

At the baseline of the study, blood samples were drawn from each participant at baseline. A total of 40 cc of whole blood was carefully processed and cultured with a specific culture medium enriched with Granulocyte-Macrophage Colony-Stimulating Factor (GM-CSF) and Interleukin-4 (IL-4) was used for a period of five days. This culture process facilitated the differentiation of the peripheral blood mononuclear cells into dendritic cells. Following the differentiation phase, the dendritic cells were further incubated for an additional two days to promote their maturation. Upon achieving the desired level of maturation, the autologous dendritic cells were then transfered through injection, subcutaneoulsy into the participant’s arm.

### 2.5. Laboratory Testing and MRI DTI

The biomarkers MMP-9 and ICAM-1 were quantified utilizing a sandwich enzyme-linked immunosorbent assay (ELISA) kit (Reed Biotech Ltd., Wuhan, China). For the DTI MRI imaging analysis, a MAGNETOM Vida MRI scanner (Siemens, Erlangen, Germany) was utilized in this study. DTI MRI examination was performed on both kidneys of the subject. Then the subject was subjected to 3 ROIs in each kidney. The three ROIs in each kidney were selected to represent the kidney’s upper, middle, and lower poles, respectively. These ROIs were chosen by a single highly trained technician under the supervision of a senior radiologist. The technician was unaware of the ROI selection results before the intervention when selecting the ROIs for the DTI MRI after the intervention, ensuring unbiased selection. After obtaining the FA value. In this study, FA was taken, with a decrease and increase in FA in patients.

MRI Scanner Specifications as follows, the MAGNETOM Vida magnetic resonance imaging (MRI) scanner, identified by serial number 176241 and material code 11060815, was utilized in this study. Operating at a magnetic field strength of 3 Tesla (which is typical for MAGNETOM Vida models), the scanner was configured for high-resolution imaging. Imaging parameters included a field of view (FoV) with a reading FoV of 250 mm and a phase FoV of 81.3%. Thirty slices were acquired with each slice having a thickness of 1.8 mm. The repetition time (RT) was set to 5800 ms, and the echo time (ET) was 71 ms, with the phase encoding direction set to horizontal (H) to forward (F). Furthermore, phase oversampling was applied at 50%.

For the DTI analysis, multi-directional diffusion weighting (MDDW) was employed with a monopolar diffusion scheme across six distinct directions. The b-values used were b = 0 s/mm^2^ with five averages and b = 330 s/mm^2^ with twelve averages. The slice thickness remained consistent at 1.8 mm, while the ET and RT were maintained at 71 ms and 5800 ms, respectively. The imaging coil elements employed were of type SP2-4 to ensure optimal data acquisition.

Post-processing involved tensor-based analysis to generate FA maps, incorporating active noise correction, dynamic field correction, and noise masking to improve the accuracy of the results.

### 2.6. Statistics

Normality was tested using Shapiro-Wilk for samples <50 and Kolmogorov-Smirnov for samples >50. FA DTI variables were analyzed with a paired *t*-test, and non-normal variables with the Wilcoxon signed-rank test. Linear regression assessed the impact of autologous dendritic cell transfer on MMP-9 and TGF β.

## 3. Results

### 3.1. Subject Characteristics

Table 1 presents the study participants’ characteristic. Twenty five subjects who met the inclusion and exclusion criteria participated in the study and adhered to the research procedures throughout all stages. The participants had an average age of 63 years, ranging from 50 to 78 years. The group included 10 men and 15 women, with 13 diagnosed with microalbuminuria and 12 with macroalbuminuria. Hypertension was the most prevalent comorbidity, affecting 24 participants (96%).

Additionally, the majority of participants were classified as overweight, with 11 subjects (44%) falling into this category based on their body mass index (BMI). The majority of participants (48%) were in stage 3 of chronic kidney disease (CKD), with 12 subjects diagnosed at this level.

### 3.2. MRI Analysis of DTI, MMP-9, and ICAM-1

**Figure 2:**
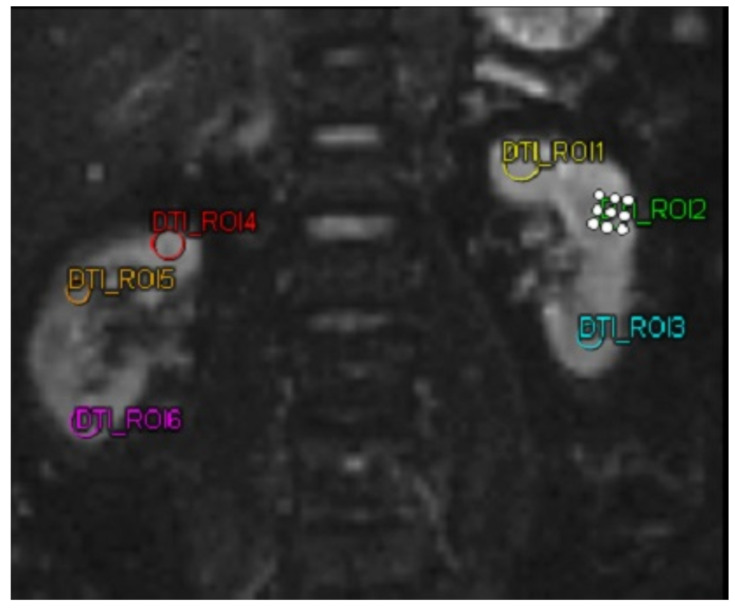
MRI DTI examination on a patient using 6 different region of interests. Abbreviations: MRI-DTI: Magnetic Resonance Imaging-Diffusion Tensor Imaging.

**Figure 3:**
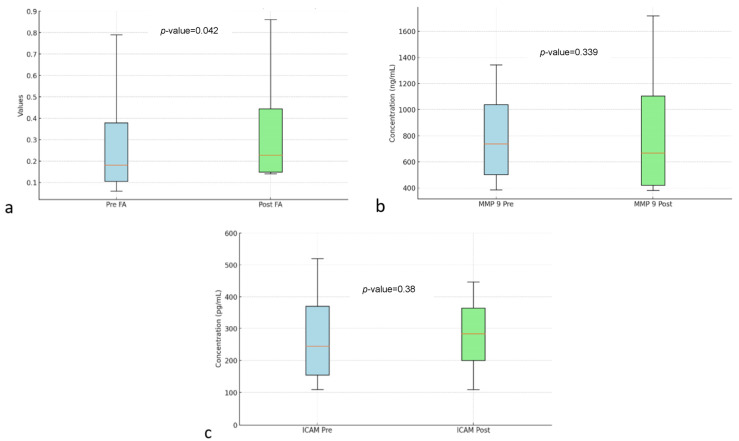
Box and whisker FA (**a**) MMP-9 (**b**) ICAM (**c**) before and after autologous dendritic cell transfer. Abbreviations: FA: Fractional Anisotropy, MMP-9: Matrix Metallopeptidase 9, ICAM: Intercellular Adhesion Molecule.

Table 2 shows the analysis of FA, MMP-9, and ICAM-1. In DTI MRI measurements (FA) before and after transfer of autologous dendritic cells (Figure 2), the median value before transfer was 242.57 ± 63.97. After transfer of autologous dendritic cells, the median increased to 305.61 ± 152.32. Statistical tests showed a significant difference with a *p*-value = 0.042. Figure 3a shows box and whisker FA on DTI MRI.

Figure 3b shows the box and whisker MMP-9. MMP-9 biomarker, the mean value before intervention was 873.68 ± 306.32. After the intervention, the mean value increased to 921.97 ± 333.33. However, no statistically significant difference was found with a *p*-value = 0.339.

Figure 3c shows box and whisker ICAM-1. ICAM-1 biomarker, the mean before intervention was 321.74 ± 84.83 with a median of 320.2 ± 109.9. After transfer of autologous dendritic cells, the mean increased to 331.8 ± 61.05. However, no statistically significant difference was found with a *p*-value = 0.38.

Table 3 illustrates the relationships between the study variables. A significant negative correlation was observed between FA and MMP-9, with a correlation coefficient (r) of −0.347 and a *p*-value of 0.016. This suggests that FA increase is associated with a reduction in MMP-9 levels. In contrast, the relationship between FA and ICAM-1 was statistically not significant, with a *p*-value of 0.402. Similarly, no significant correlation was found between MMP-9 and ICAM-1, with a *p*-value of 0.409.

## 4. Discussion

This study revealed that hypertension was the most prevalent comorbidity among the research participants. Hypertension plays a role in exacerbating DKD by increasing intraglomerular pressure. DM and hypertension cause synergistic effects that increase endoplasmic reticulum stress and mitochondrial dysfunction in glomerular cells [14]. DKD associated with hypertension results from a chronic inflammatory reaction, which causes glomerular capillary injury and decreased renal function. This chronic inflammation will release pro-inflammatory cytokines, contributing to increased blood pressure and fibrosis in the kidney [15].

Based on the research results presented above, it can be seen that therapy using autologous dendritic cells has different effects on the various parameters measured. On MRI DTI FA, there was a significant improvement after therapy with a *p*-value of 0.042, indicating a positive change in tissue structure or function measured through MRI DTI FA imaging. This indicates that the therapy may have contributed to the improvement. FA in the kidney is related to the use of DTI sequences in renal imaging, which aims to assess the diffusion of water molecules in kidney tissue. FA is one of the important parameters in DTI that measures the direction and extent of water diffusion anisotropy, which is related to kidney structure and function. The use of DTI MRI in the kidney has shown that FA can be used as an indicator of microstructural damage, such as fibrosis or changes in the renal tubules. In the context of Diabetic Kidney Disease, FA measurement could be an important diagnostic tool to detect microstructural changes that cannot be seen through conventional imaging, enabling earlier detection and assessment of response to therapy [16]. FA values exhibit a negative correlation with serum creatinine and cystatin C, while showing a positive correlation with eGFR [7].

The underlying mechanism through which autologous dendritic cell transfer improves FA in DKD patients involves immune modulation, inflammation reduction, and potential kidney tissue repair. Dendritic cells, essential for immune responses, can be genetically modified to boost their immune-regulatory functions, thereby fostering tolerance and mitigating inflammation [13]. In DKD, chronic inflammation plays a significant role in kidney damage, contributing to fibrosis, endothelial dysfunction, and tubular injury [15]. By modulating the immune response, dendritic cells help reduce this inflammatory state, leading to improvements in kidney tissue integrity, as reflected by the increase in FA [6]. FA measures the integrity of kidney microstructure, with higher values indicating better tissue organization and function [7]. This improvement suggests that dendritic cell therapy may aid in kidney tissue repair by reducing fibrosis, enhancing podocyte function, and restoring the alignment of renal microstructures [17]. Ultimately, the increase in FA observed after dendritic cell transfer likely reflects the regenerative effects of the therapy, which promotes kidney repair through immune modulation and inflammation reduction.

This study also found a significant correlation between FA and inflammatory biomarker MMP-9 (*p* = 0.025). This finding is consistent with the study by Seah et al., which identifies a relationship between FA and inflammatory biomarkers [18]. In the early stages of DKD, MMP-9 increases as an initial response to hyperglycemia and oxidative stress. Increased MMP-9 is involved in tubular basement membrane degradation and inflammatory cell recruitment, leading to damage to the kidneys [19]. This study shows that an increase in FA is associated with a decrease in MMP-9. A decrease in MMP-9 can improve kidney function in DKD. Decreased MMP-9 can modulate podocyte function and inhibit dedifferentiation. Suggesting that lower MMP-9 may contribute to improved renal function by maintaining podocyte integrity and function, which is important in maintaining glomerular filtration [20]. MMP-9 is also positively associated with albuminuria, so a decrease in MMP-9 indicates a decrease in albuminuria [20].

In this study, there were changes in MMP-9 values that were not significant. Changes in MMP-9 are likely due to the longer follow-up time required in this study and the multiple transfer of autologous dendritic cells. This is consistent with the study by Miyazaki et al., which examined the changes in MMP-9 levels, noting a significant decrease within 12 months following the administration of ACE inhibitors (ACEi) or angiotensin II receptor blockers (ARB) during the same period [21].

This study found no significant alterations in ICAM-1 values. Future studies are expected to assess ICAM-1 once a week for one month. Research conducted by Shukla et al. assessed ICAM-1 seven days after the intervention [22]. Further research is expected to assess ICAM-1 once a week for seven days to assess changes in ICAM-1.

In this study, there were significant changes in FA on DTI MRI, while the biomarkers MMP-9 and ICAM-1 showed no significant changes. This may be because there are other inflammatory biomarkers, which can affect the value of FA. Research conducted by Tripathi et al. showed a significant correlation of FA with IL-1β and TNF α [23]. In research conducted by Rodrigue et al., there is a significant relationship between FA with IL 8 and TNF α [24].

## 5. Conclusions

Autologous dendritic cells transfer significantly enhanced FA in kidney tissue, suggesting its potential to improve kidney function in DKD. However, no significant changes in inflammatory biomarkers like MMP-9 and ICAM-1 underscores the complexity of immune modulation in DKD, indicating that multiple therapeutic mechanisms may be involved. While the results are promising, additional research with a larger sample size, randomized control groups, and extended follow-up periods is crucial to further validate the efficacy of dendritic cell therapy. Future studies should also investigate the potential synergistic effects of combining dendritic cell therapy with other treatments to optimize therapeutic outcomes. Moreover, expanding the range of biomarkers and imaging modalities, such as DTI with molecular imaging, could offer a more thorough understanding of the treatment’s impact on kidney structure and function.

## Figures and Tables

**Table 1:** Subject’s Characteristics.

		Count	Table N %
Age	<60	9	36.0%
	≥60	16	64.0%
Gender	Women	15	60.0%
	Men	10	40.0%
UACR	Microalbuminuria	13	52.0%
	Macroalbuminuria	12	48.0%
Chronic Kidney Disease Stage	1	9	36.0%
	2	4	16.0%
	3	12	48.0%
Hypertension	No	1	4.0%
	Yes	24	96.0%
Neuropathy	No	8	32.0%
	Yes	17	68.0%
Heart Disease	No	14	56.0%
	Yes	11	44.0%
Stroke	No	23	92.0%
	Yes	2	8.0%
Insulin	No	7	28.0%
	Yes	18	72.0%
Sulphonylurea	No	19	76.0%
	Yes	6	24.0%
Biguanide	No	21	84.0%
	Yes	4	16.0%
Thiazolidinedione (glitazone)	No	24	96.0%
	Yes	1	4.0%
Glinid	No	25	100.0%
	Yes	0	0.0%
SGLT2-i (glifozin)	No	17	68.0%
	Yes	8	32.0%
GLP-1 agonist	No	25	100.0%
	Yes	0	0.0%
DPP4-i (gliptin)	No	20	80.0%
	Yes	5	20.0%
α-glucosidase-i	No	23	92.0%
	Yes	2	8.0%
Central a-agonist (clonidine)	No	24	96.0%
	Yes	1	4.0%
Diuretic	No	22	88.0%
	HCT	3	12.0%
	Spironolactone	0	0.0%
a-blocker	No	25	100.0%
	Yes	0	0.0%
CCB	No	8	32.0%
	DHP	13	52.0%
	Non-DHP	4	16.0%
ACE-i	No	22	88.0%
	Yes	3	12.0%
b-blocker	No	15	60.0%
	Yes	10	40.0%

Abbreviations: ACE-i: Angiotensin Converting Enzyme Inhibitor, GLP-1: Glucagon-Like Peptide-1, SGLT2-i: Sodium-glucose Cotransporter-2 Inhibitor, HCT: Hydrochlorothiazide, CCB: Calcium Channel Blocker, DPP4-i: Dipeptidyl Peptidase-4 Inhibitor, UACR: Urinary Albumin-Creatinine Ratio, DHP: Dihydropyridine.

**Table 2:** Analysis of FA, MMP-9, and ICAM before and after transfer of autologous dendritic cells.

		Before Autologous Dendritic Cells	After Autologous Dendritic Cell	*p*-Value
MRI DTI	FA (Median ± IQR)	0.242 ± 0.063	0.305 ± 0.152	0.042
Biomarkers	MMP-9 (Mean ± SD) ng/mL	873.68 ± 306.32	921.97 ± 333.33	0.339
	ICAM (Mean ± SD) pg/mL	321.74 ± 84.83	331.8 ± 61.05	0.38

Abbreviations: MRI DTI: Magnetic Resonance Imaging Diffusion Tensor Imaging, FA: Fractional Anisotropy, IQR: Interquartile Range, MMP-9: Matrix Metallopeptidase 9, SD: Standard Deviation, ICAM: Intercellular Adhesion Molecule.

**Table 3:** Relationship between variables in the study.

Variables	FA (r, *p*)	MMP-9 (r, *p*)	ICAM (r, *p*)
FA	-	(−0.347, 0.016)	(−0.124, 0.402)
MMP-9	(−0.347, 0.016)	-	(−0.122, 0.409)
ICAM	(−0.124, 0.402)	(−0.122, 0.409)	-

Abbreviations: FA: Fractional Anisotropy, MMP-9: Matrix Metallopeptidase 9, ICAM: Intercellular Adhesion Molecule, r: Correlation coefficient, *p*: *p*-value.

## Data Availability

Data is unavailable due to privacy or ethical restrictions.
